# Willingness to donate eyes and its associated factors among adults in Gondar town, North West Ethiopia

**DOI:** 10.1186/s12886-017-0577-1

**Published:** 2017-10-02

**Authors:** Mohammed Seid Hussen, Kbrom Legesse Gebreselassie, Asamere Tsegaw Woredekal, Nebiyat Feleke Adimassu

**Affiliations:** 1 Department of Optometry, College of Medicine and Health Sciences, University of Gondar, Gondar, Ethiopia; 2 Department of Ophthalmology, College of Medicine and Health Sciences, University of Gondar, Gondar, Ethiopia

**Keywords:** Willingness, Eye donation, Gondar, Ethiopia

## Abstract

**Background:**

In Ethiopia, there is a substantial mismatch between need and supply of corneal transplant. Although corneal transplantation service is affected by various factors, willingness to donate eyes is an essential indicator of its availability, accessibility, and acceptability. Therefore, this study aimed to determine the magnitude of willingness to donate eyes and its associated factors, which help to develop appropriate strategies that can address this undersupply and unmet need.

**Methods:**

A community-based cross-sectional survey was conducted on 774 adults who were selected using multistage random sampling in Gondar town, North West, Ethiopia. The data were collected through interviews.

**Results:**

In this survey, 774 adults with a median age of 30 ± 14.33 years participated. The proportion of willing to donate eyes was 37.6% [95% CI: 34.3%–41.3%]. It was positively associated with the religious belief of Christianity [AOR = 1.73, 95% CI: 1.08–2.75], having awareness about eye donation [AOR = 1.38, 95% CI: 1.01–1.92], educational level of high school [AOR = 2.90, 95% CI: 1.72–4.90], and College/University [AOR = 2.23, 95% CI: 1.28–3.87].

**Conclusion:**

The magnitude of willingness to donate eyes was moderate and positively associated with the higher educational level and awareness. It is, therefore, strategic to plan awareness creation programs to mobilize the community.

**Electronic supplementary material:**

The online version of this article (10.1186/s12886-017-0577-1) contains supplementary material, which is available to authorized users.

## Background

Globally, corneal blindness is the second most prevalent ocular condition, especially in developing countries. Bilateral corneal blindness accounts for 12% (4.9 million) of 39 million blind [1, 2]. In Ethiopia, 19.3% of all blind cases are contributed by corneal blindness. It mainly results from trachoma, Xerophthalmia, use of harmful traditional eye medicines, onchocerciasis and ocular trauma [2, 3]. Ultimately, the condition results in loss of productivity for those of young and middle age adult [4].

The best option to rehabilitate the impaired vision is a corneal transplantation worldwide. However, the potentially limiting factor in planning transplantation is the shortage of donated corneas. It is also aggravated by the availability of inefficient domestic eye bank, lack of potential donors and weak cooperation of close relatives to collect pledged cornea [5].

In developing countries, where the magnitude of corneal blindness is higher, the availability of donated corneas is very low. In Ethiopia, between 130 and 150 corneas are harvested yearly [5]. However, there are more than 300,000 blind people due to corneal disease. This depicts that there is a potential gap between demand and supply of corneal graft.

Some studies revealed that willingness to donate eyes and close relatives ‘cooperativeness to donate pledged eyes is essential to ensure the corneal transplantation service [5]. In developed countries, the decision to be an organ donor is affected by relational ties, religious beliefs, previous exposure to a health care, cultural and family influences [6].

A study was done regarding willingness to donate eyes in central Ethiopia. However, the demographic distribution of central Ethiopia with respect to religion, cultural and social values is different from Northern Ethiopia. Thus, it is difficult to generalize this study. This indicates that there is a limitation of evidence regarding willingness to donate eyes in the area. It was, therefore, important to determine the magnitude of willingness to donate eyes and its associated factors. The finding of this study helps to develop strategies that can address this undersupply and unmet need.

## Methods

### Study population and study design

A community-based cross-sectional design study was employed. The study was conducted in Gondar town, North West Ethiopia, March 13, 2016. All adults aged ≥18 years had equally likely chance to participate in this survey. Nevertheless, those adults who had corneal blindness in both eyes, and mental illness were excluded.

### Sample size determination

The entire sample size was 825, which was determined using the single population proportion formula. During computing the sample size, 95% confidence level, the proportions of willing to donate eyes from a similar study conducted in central Ethiopia (57.9%) [7], 5% margin of error and 10% for non-response rate were assumed. Then the computed sample size was multiplied by the design effect of two for ensuring its representativeness. The study participants were selected using a multistage random sampling method.

### Data collection tool and procedures

A structured hard copy questionnaire was utilized. It was derived from previous studies [7, 8]. The questionnaire comprised of 25 questions regarding knowledge about eye donation, willingness to donate eyes, and socio-demographic characteristics (Additional file 1). The questionnaire was first prepared in English language, then translated to Amharic and later back translated into the English language to maintain its consistency. The data were collected by trained optometrists through face to face interviews.

**Willingness to donate eyes** was categorized as **willing** if a subject had the interest to pledge to donate his/her eyes and **unwilling** if a subject was involuntary to pledge to donate his/her eyes or not decided.

**Knowledge** was also assessed by using 11 questions. Each correct response had a score of 1 and each wrong or ‘do not know’ response had a score of 0. The sums of scores varied from 0 to 11 points. Finally, the overall knowledge was categorized using modified Bloom’s cut off points as low, moderate and high if the scores were <50% (0–5 points), between 50 and 79% (6–7 points) and from 80 to 100% (8–11 points), respectively [9].

### Data processing and statistical analysis

After the data were checked for completeness, it was entered into EpiData version 3. Then it was exported to SPSS version 20. The descriptive parts of the data were summarized using measures of central tendency and dispersion. Associated factors for willingness to donate eyes were identified by using Binary logistic regressions. Thus, statistically significant association was considered for those factors with a *p*-value of less than 0.05. The analyzed data were organized and presented in a tabular and graphical form as per necessity.

### Ethical consideration

Ethical approval was obtained from the School of Medicine; Ethical review committee, University of Gondar. The objective of the study was clearly described for each participant. Then written informed consent was received from each study participant. The survey participants were granted full right to cease or refuse to take part. Confidentiality was assured through keeping records and omitting any participants’ identifiers.

## Results

### Socio-demographic and economic characteristics of the study subjects

In this study, the median age of the study participants was 30 ± 14. 33 years. Among 774 participants, 64.7% of them were females. The majority of the respondents were Christian (85.6%) followed by Muslims (14.4%). Thirty-five percent of the study participants have completed high school (Table 1).

**Table 1:** Socio-demographic and economic characteristics of study participants, Gondar town, North West, Ethiopia, 2016 (*n* = 774)

Variables	Frequency	Percent
Age(year)		
18–30	403	52.1
31–43	181	23.4
≥44	190	24.5
Sex		
Female	501	64.7
Male	273	35.3
Religion		
Christianity	663	85.6
Islam	111	14.4
Ethnicity		
Amhara	711	91.9
Tigrayan	39	5
Qemant	24	3.1
Marital status(currently)		
Married	401	51.8
Single	274	35.4
Divorced	52	6.7
Widow	47	6.1
Educational level		
No formal education	114	14.8
Primary school	121	15.6
High school	273	35.3
College/university	266	34.3
Occupation		
Non health care professionals	757	97.8
Health care professionals	17	2.2
Monthly income(Birr)		
≤1000	275	35.5
1000–2000	180	23.3
2000–3000	143	18.5
> 3000	176	22.7

**Education categories**: No formal education (can read and write or not), primary school (1-8th), high school (8-12th), and college/university (diploma, degree, and above)

**Income categories**: Income was categorized based on country per capita income per month of Ethiopia (997 Ethiopian Birr), as follow, ≤1000**,** 1000–2000**,** 2000–3000 and >3000

### Participants’ knowledge about eye donation

**Fig. 1:**
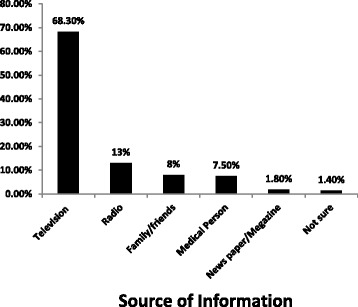
Source of information for eye donation among adults in Gondar town, North West, Ethiopia, 2016

Around 57% of the participants heard about eye donation before [95% CI: 53.2% -60.2%]. Their main source of information was a television (68.33%) (Fig. 1). The median knowledge score point for those participants who had awareness was 6.0 ± 1.76 points. Overall, 23.7% of the participants had a good level of knowledge about eye donation [95% CI: 19.7%–27.7%] (Table 2).

**Table 2:** Participants’ awareness and knowledge about eye donation among adults in Gondar town, North West Ethiopia, 2016

Variables	Frequency	Percent	95% CI
Awareness (*n* = 774)			
Yes	439	56.7	[53.2% -60.2%]
No	335	43.3	[39.8%–46.8%]
Level of Knowledge (*n* = 439)			
Low	145	33	[28.3%–37.7%]
Moderate	190	43.3	[38.7%–47.9%]
Good	104	23.7	[19.7%–27.7%]

### The proportion of willingness to donate eyes

**Fig. 2:**
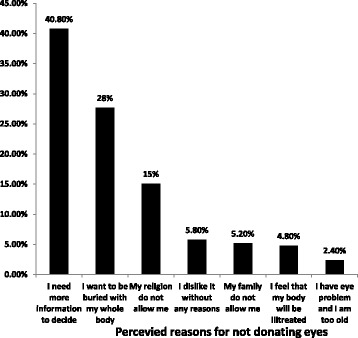
Participants’ Perceived reasons for not donating their eyes among adults in Gondar town, North West, Ethiopia, 2016

Among the total of 774 participants, 37.6% of them were willing to donate their eyes [95% CI: 34.2%–41.1%]. The majority of the study participants (73.3%) were also willing to donate pledged close relatives’ eyes upon the death of a person. Having a thought of that ‘eye donation is pleased (69.75%) and noble human act (19.7%) were the primary reasons given for being willing to donate eyes (Table 3). Nevertheless, the most prevalent reasons for not donating their eyes were requiring further information to decide (40.8%); desiring to be entombed with their whole body (25.7%) and religious restriction (15.1%) (Fig. 2).

**Table 3:** Willingness to donate eyes and perceived reasons, among adults in Gondar town, North West, Ethiopia, 2016 (*n* = 774)

Variables	Frequency	Percent	95% CI
Willingness to donate own eyes			
Yes	291	37.6	[34.2%–41.1%]
No	483	62.4	[59.98%–65.8%]
Willingness to donate close relatives’ eyes			
Yes	567	73.3	[70.2%–76.4%]
No	207	26.7	[23.6% - 29.8%]
Reasons for being willing to donate eyes (*n* = 291)			
It is pleased to help blind person	203	69.75	[64.5%–75.1%]
Eye donation is noble work	57	19.60	[15.0%–24.2%]
It is both noble and a pleasure activity	23	7.90	[4.8%–11.0%]
My eyes are not useful after my death	8	2.75	[0.95%–4.62%]

### Associated factors of willingness to donate eyes

In a multivariable binary logistic regression, only religion, educational level, and awareness were associated with willingness to donate eyes. Thus, those participants who had the educational status of the high school, and College/University were 2.90 and 2.23 times more likely to be willing to donate their eyes than those who had no formal education, respectively. In addition, those who had awareness about eye donation were 1.38 times more likely to be willing to donate their eyes than those participants who had no awareness [AOR = 1.38, 95% CI: 1.01–1.92] (Table 4).

**Table 4:** Binary logistic regression output showing the effect of different variables on willingness to donate own eyes among adults, Gondar town, Northwest Ethiopia, 2016

Variables	Willingness to donate eyes			
	Yes	No	Crude OR (95% CI)	Adjusted OR (95% CI)
Age (year)				
18–30	157	246	1.35 (0.94, 1.940)	1.04 (0.65, 1.63)
31–43	73	108	1.43 (0.93, 2.18)	1.09 (0.68, 1.76)
≥44	61	129	1.00	1.00
Marital status				
Single	106	168	1.00	1.00
Married	157	244	1.02 (0.74, 1.39)	1.11 (0.72, 1.59)
Divorced	16	36	0.7 (0.37, 1.33)	0.92 (0.45, 1.85)
Widow	12	35	0.54 (0.27, 1.09)	1.01 (0.44, 2.33)
Ethnicity				
Amhara	260	451	1.00	1.00
Tigrayan	18	21	1.5 (0.78, 2.84)	0.55 (0.23, 1.28)
Qemant	13	11	2.1 (0.91, 4.64)	0.81 (0.27, 2.35)
Religion				
Christianity	262	401	1.85 (1.178, 2.90)	1.73 (1.08, 2.75)*
Islam	29	82	1.00	1.00
Educational level				^a^**
No formal education	24	90	1.00	1.00
Primary school	33	88	1.41 (0.77, 2.57)	1.39 (0.76, 2.57)
High school	122	151	3.03 (1.82, 5.04)	2.90 (1.72, 4.90)**
College/university	112	154	2.73 (1.64, 4.55)	2.23 (1.28, 3.87)*
Monthly income(birr)				
≤1000	97	178	1.00	1.00
1000–2000	61	119	0.94 (0.63, 1.39)	0.77 (0.51, 1.18)
2000–3000	47	96	0.89 (0.58, 1.37)	0.69 (0.44, 1.10)
> 3000	86	90	1.75 (1.19, 2.58)	1.34 (0.86, 2.05)
Awareness				
Yes	186	253	1.61 (1.19, 2.17)	1.38 (1.01, 1.92)*
No	105	230	1.00	1.00

*0.001 < *p*-value <0.05 ** *p*-value <0.001 a: overall *p*-value

## Discussion

The proportion of willing to donate their eyes was 37.6%, which is higher than the studies done in Melaka, Malaysia [8], and Ghana [10]. However, this result is lower than the studies done in South India [11], Australia [12], Singapore [13, 14], and Ethiopia [7]. The difference might be aroused from variation in literacy level, awareness level, availability of eye banks and study setting.

It was also found that having a thought of that ‘eye donation is pleased (69.75%) and a noble act (19.7%) was the main reasons given for being willing to donate eyes. Similar results were reported from previous studies done in Singapore [13] and central Ethiopia [7]. It indicates that a person’s altruistic values to achieve a desired goal of life will matter a person’s decision to donate eyes.

Since the decision to be an organ donor requires detail information about religious doctrine, cultural and social values [6], 40.8% of unwilling participants required additional information to make their decision. This finding depicts that maximum efforts need to be exerted to provide a detailed information about eye donation to shape their attitude. The next cited reasons for not willing to donate their eyes were having a thought of to be buried with their whole body (25.7%) and religious restriction (15.1%). The origin of such reasons may be related to religious or cultural values. It was likewise expressed in a way that cultural and religious beliefs may be interchangeable and some people may hold culturally specific beliefs, which are not bound to any particular religious doctrine [6].

In a multivariable binary logistic regression, religion, educational level, and awareness were associated with the willingness to donate eyes. Thus, those participants who had the educational status of the high school, and College/University were 2.90 and 2.23 times more likely being willing to donate their eyes than those who had no formal education, respectively. Similar findings were reported from studies done in Nanjing, China [15], Ghana [10] and Jimma University, Ethiopia [4]. This association may be because individuals with higher educational status may have the favorable knowledge and attitude towards organ donation. This explanation is more supported by a study done in Washington, USA [16]. However, a study from Melaka, Malaysia showed that educational status is not associated with the willingness to donate eyes [8]. These studies were institutional based. This may be the possible reason for the argument.

The association found between willingness to donate eye and awareness regarding eye donation is in accord with previous surveys done in Central Ethiopia [7], Jimma University, Ethiopia [4] and Singapore [13]. This result implicates that providing comprehensive information about eye donation is essential to motivate the community.

This study also found an association between willingness to donate eye & religion of Christianity as compared to the religion of Islam. This result in line with studies from Singapore [13] and India [17]. Furthermore, this finding is backed up by a qualitative study done at Oxford University, USA [6].

In the present study, there was no any association between age and willingness to donate eyes. Studies conducted in India, Singapore, and Nanjing, China reported that willingness to donate eyes was significantly higher among the older age population [14, 18, 19]. Nevertheless, a study performed in urban India showed that willingness to donate eyes was significantly lower in older adult [17]. This dispute might be due to variation in age categorization.

Since the study is a community-based research with a large sample, a generalization to the target population is not questionable. It would also be highly magnificent if it had a qualitative component.

## Conclusions

The magnitude of willingness to donate eyes found from this study was moderate. It was also found that educational status; awareness and religion were identified as a statistically significant factor. It is, therefore, strategic to plan awareness creation programs to mobilize the community.

## Additional file

Additional file 1: English version of structured questionnaires. (DOC 73 kb)
