# Supplementary material for: Willingness to donate eyes and its associated factors among adults in Gondar town, North West Ethiopia
Source: BMC Ophthalmol. 2017 Oct 2;17:178. doi: 10.1186/s12886-017-0577-1 (PMC5625743; doi:10.1186/s12886-017-0577-1)
Supplement: Additional file 1: — English version of structured questionnaires. (DOC 73 kb) [file 12886_2017_577_MOESM1_ESM.doc]

# English version of structured questionnaires

Pretested structured questionnaire to determine the proportion of willingness to donate eyes and associated factors among adults in Gondar town, North West Ethiopia.

Name of Kebele------------------

**Code number---------------------**

| **Section 1 :Participants’ socio-demographic data** | | | | | | |
| --- | --- | --- | --- | --- | --- | --- |
| S.no | | | Questions | | | Response options |
|  | | | Your age | | | _______________________in year |
|  | | | Sex | | | 1. Male 2. Female |
|  | | | Religion | | | 1. Orthodox 2. Muslim 3. Protestant 4. Catholic 5. Other ____________ |
|  | | | Ethnicity | | | 1. Amhara 2. Tigrayan 3. Qemant 4. Oromo 5. Others___________ |
|  | | | Marital status | | | 1. Single 2. Married 3. Divorced 4. Widow |
|  | | | Completed educational level | | | 1. No formal education 2. Primary school 3. High school 4. College /University |
|  | | | Your occupation | | | _________________________________ |
|  | | | Family monthly income | | | ___________________________Birr |
| **Section 2: Participants’ awareness about eye donation** | | | | | | |
|  | | Have you ever heard about eye donation? | | | | 1. Yes 2. No |
|  | | If your answer is Yes in Q1, what is your source of information? | | | | 1. Medical personnel 2. Newspapers/Magazine 3. Television 4. Radio 5. Family member/Friends 6. Other______________________________ |
|  | | If the participants have no any information about eye donation, please describe what eye donation mean and then skip to section 4 (for data collector: Eye donation is the act of donating eyes after one’s death for someone to see again) | | | | |
| **Section 3: Participants’ knowledge about eye donation** | | | | | | |
| 1. . | | | If your answer is Yes in Q1, What do you mean by eye donation? | 1. Donation of eyes after one’s death 2. Help someone to see again by donating eyes 3. Giving corneas to a blind person after one’s death 4. Others_____________ 5. Do not know | | |
|  | | | Can eyes be removed from a living person for a donation? | 1. Yes 2. No 3. Don’t know | | |
|  | | | Can living person pledge to donate his or her eyes? | - 1. Yes   2. No   3. Don’t know | | |
|  | | | Is it mandatory to get the consent of family members for eye donation after the death of the person? | 1. Yes 2. No 3. Don’t know | | |
|  | | | What is removed from the donor's eye? | 1. Whole eye ball 2. Cornea 3. Lens 4. Others------------- 5. Don’t know | | |
|  | | | What is the ideal time duration to retrieve eyes after the death of the person? | 1. As soon as possible 2. Within 6 hours 3. Within 48 hours 4. Others____________ 5. Don’t know | | |
|  | | | Can a person who wearing spectacles donate eyes? | 1. Yes 2. No 3. Don’t know | | |
|  | | | Can a person with HIV donate his/her eyes? | 1. Yes 2. No 3. Don’t know | | |
|  | | | Do you think eye donation can cure all types of blindness? | 1. Yes 2. No 3. Don’t know | | |
|  | | | Is there any eye bank found in Ethiopia? | 1. Yes 2. No 3. Don’t know | | |
|  | | | Do you think that a human eye can be bought or sold? | 1. Yes 2. No 3. Don’t know | | |
| **Section 4: Participants’ willingness to donate eyes** | | | | | | |
|  | Are you willing to donate your eyes? | | | | 1. Yes 2. No 3. I need time/more information to decide | |
|  | Are willing to donate close relative’s eye if he/she had pledged to donate eye? | | | | 1. Yes 2. No 3. I need time/more information to decide | |
|  | If your answer is yes, what is your perceived reason willing to donate? | | | | 1. It is pleasure to help a blind person 2. Eye donation is noble work 3. Eye donation is both a pleasure and noble act 4. My eyes are not useful after my death 5. Other_________________________________________ | |
|  | If your answer is No, what is your perceived reason not willing to donate? | | | | 1. I need more information to decide 2. Family members object to eye donation 3. It is against my religion 4. Feels that body is ill-treated by eye donation 5. I want to be my body intact after death 6. I have eye problems 7. I am too old 8. Other------------------------------------------- | |
